# Effect of primer composition on zirconia surface properties and bond strength

**DOI:** 10.1111/eos.70052

**Published:** 2025-11-24

**Authors:** Otavio Marino dos Santos Neto, Ingrid Carneiro Cavalcante Souto, Ana Paula Macedo, Rossana Pereira de Almeida

**Affiliations:** ^1^ School of Dentistry of Ribeirão Preto University of São Paulo (FORP–USP) Ribeirão Preto São Paulo Brazil

**Keywords:** adhesives, dental materials, permanent dental restoration, resin cements, wettability

## Abstract

This study investigated the effects of different surface treatments on surface roughness, wettability, and shear bond strength of 3Y‐TZP (yttria‐stabilized tetragonal zirconia polycrystal). A total of 180 specimens (6 × 6 × 2 mm) underwent sandblasting with 50 µm Al_2_O_3_ and were divided into six groups: (i) control (no treatment), (ii) 10‐MDP + silane primer, (iii) universal adhesive with 10‐MDP and HEMA, (iv) silane‐based primer with phosphate methacrylate, (v) methacrylate‐based primer with ethanol, and (vi) PMDM primer. Surface roughness and wettability were measured before and after treatment. For shear bond strength evaluation, each group was subdivided by type of resin cement. After 10,000 thermal cycles, shear bond strength and adhesive remnant index were measured. Surface treatments increased roughness and wettability. Primers with 10‐MDP and silane had the highest bond strength, followed by silane‐based primer and universal adhesive. Primers with other functional monomers showed intermediate performance, while the control and PMDM primer had the lowest values. Adhesive remnant index analysis revealed predominantly type 1 failures for 10‐MDP + silane and silane‐based primers, whereas universal adhesive and other primers showed mixed failures. These results indicate that combining sandblasting with functional surface agents improves zirconia adhesion, with primer composition influencing long‐term bonding effectiveness.

## INTRODUCTION

The search for esthetic and durable materials has driven the transition from metal‐ceramic systems to metal‐free alternatives in both conventional and implant‐supported fixed prostheses. In recent years, zirconia has been widely used due to its exceptional mechanical properties and satisfactory esthetics [1]. Technological advancements have led to the development of different zirconia generations, each with distinct compositions and microstructures, directly affecting strength, translucency, and clinical indications [2, 3, 4].

First‐generation zirconia was predominantly opaque, with high mechanical strength but low translucency, limiting its use to posterior regions. The second generation improved translucency while maintaining good strength, expanding its suitability for esthetic restorations. The third generation further prioritizes translucency, albeit with slightly reduced mechanical resistance, and is mainly indicated for single crowns in esthetic areas [2, 4]. Third‐generation zirconia has become widely used for single‐unit restorations due to its combination of esthetic and adequate mechanical properties, allowing broader clinical application [2, 4]. However, zirconia still presents challenges regarding bonding to luting agents—a critical factor for restoration longevity [5, 6]. Its inert surface hinders stable chemical bonding, requiring effective surface treatment strategies to improve adhesion.

The sandblasting using aluminum oxide has been extensively studied as a conditioning method. This process increases surface roughness and forms a protective layer with embedded particles, enhancing wettability and adhesion [7, 8]. Using 50 µm particles has shown benefits without compromising mechanical strength [9, 10]. Optimizing the surface with abrasion is particularly important because it enhances the interaction between zirconia and adhesive agents, facilitating chemical bonding. Bonding agents containing phosphate monomers, such as 10‐MDP, have proven effective in creating stable adhesive interfaces with zirconia [11, 12]. The 10‐MDP molecule forms durable chemical bonds with zirconia's metal oxides, making it essential in modern adhesive protocols [13].

Combining sandblasting with 10‐MDP‐containing agents provides a synergistic approach to enhance zirconia adhesion. Surface optimization by abrasion improves the chemical interaction promoted by 10‐MDP, resulting in greater bond strength and clinical predictability [14, 15]. Studies support the effectiveness of these strategies in extending restoration longevity, even under adverse conditions [16, 17]. Despite these promising results, a standardized adhesive cementation protocol that consistently ensures optimal outcomes for zirconia has not yet been fully established [15, 18, 19]. This gap highlights the need for further research to refine surface treatment protocols for polycrystalline ceramics, aiming to optimize the interaction between ceramic, resin cement, and dental substrate over time.

However, the comparative effectiveness of newer primers and universal adhesives, specifically on third‐generation zirconia following standardized abrasion, remains less clear. In this context, the present study aimed to evaluate and compare the effectiveness of sandblasting combined with different chemical treatments in the form of bonding agent application on third‐generation zirconia. The study considered both surface characteristics and adhesive bond strength using two types of dual‐cure resin cements. The null hypothesis was that these surface treatments, combining aluminum oxide abrasion with bonding agents, do not significantly alter surface roughness or wettability compared to abrasion alone (control), nor do they affect the bond strength between zirconia, resin cement, and composite resin or the failure mode after thermocycling.

## MATERIAL AND METHODS

**FIGURE 1:**
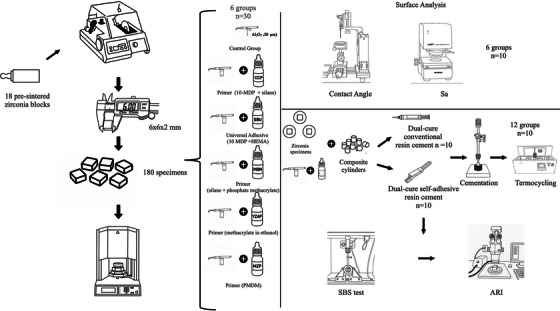
Schematic representation of the experimental workflow.

Figure 1 provides a schematic overview of the experimental workflow. Zirconia specimens were prepared from presintered zirconia blocks suitable for crowns, fixed partial dentures, and abutments (ZirkOM ST, Aidite High‐Technical Ceramics). The blocks were sectioned using a high‐precision saw (Isomet 1000 Precision Saw, Buehler) with a diamond disc under water cooling. The specimens were then sequentially polished with water sandpaper (#400, #600, #800, #1200) and ultrasonically cleaned for 5 min to eliminate contaminants. Their final dimensions were confirmed using a digital caliper. The restorative materials utilized in this study, along with their brand names, manufacturers, and chemical compositions, are detailed in Table 1.

**TABLE 1:** Commercial names, manufacturers, and compositions of the materials employed in this study.

Material	Brand name	Manufacturer	Composition
Adhesive system	Single Bond Universal	3M ESPE	10‐MDP phosphate monomer, HEMA, dimethacrylate resin, vitreous bond copolymer, ethanol, water, silane (pH: 2.7)
Ceramic primer	Monobond N	Ivoclar‐Vivadent	Ethanol, silane, phosphoric methacrylate, methacrylate sulfide
Ceramic primer	MZ Primer	Angelus	PMDM, HEMA‐P, methacrylic acid, benzoyl peroxide, and solvent (acetone)
Ceramic primer	Clearfil Ceramic Primer Plus	Kuraray Noritake	3‐methacryloxypropyl trimethoxysilane, 10‐MDP, ethanol
Ceramic primer	YZAP	Yller	Methacrylate monomers, ethanol.
Dual core self‐adhesive resin cement	Rely X U200	3M ESPE	Base paste: methacrylate monomers containing phosphoric acid groups, methacrylate monomers, silanated fillers, initiator components, stabilizers, rheological additives. Catalyst paste: methacrylate monomers, alkaline (basic) fillers, silanated fillers, initiator components, stabilizers, pigments, rheological additives
Dual core conventional resin cement	Panavia V5	Kuraray Noritake	Paste A: Bis‐GMA, TEGDMA, hydrophobic aromatic dimethacrylate, hydrophilic aliphatic dimethacrylate, initiators, accelerators, silanated barium glass, silanated fluoroalminosilicate glass. Paste B: Colloidal silica, Bis‐GMA, Hydrophobic aromatic dimethacrylate, hydrophilic aliphatic dimethacrylate, silanated barium glass, silanated aluminum oxide, accelerators, camphorquinone, pigments.
Zirconia based CAD/CAM block	Zirkom ST Super	Aidite	ZrO_2_ + HfO_2_ + Y_2_O_3_ (≥ 99%); Y_2_O_3_ ≈ 4–6%; Al_2_O_3_ ≤ 0.15%; trace oxides for coloring

Abbreviations: %, percentage; 10‐MDP, 10‐methacryloyloxydecyl dihydrogen phosphate; Ac, acetone; Al_2_O_3_, aluminum oxide; BIS‐GMA, bisphenol A‐glycidyl methacrylate; EtOH, ethanol; GPDM, glycerophosphate dimethacrylate; HEMA, hydroxyethyl methacrylate; HfO_2_, hafnium dioxide; PMDM, pyromellitic dianhydride dimethacrylate; TEGDMA, triethylene glycol dimethacrylate; Y_2_O_3_, yttrium oxide; ZrO_2_, zirconium dioxide.

The material's postsintering shrinkage percentages were precalculated to ensure a final specimen size of 6 × 6 × 2 mm. Zirconia specimens were sintered in a furnace (inFire HTC Speed, Sirona Dental) following the manufacturer's recommendations.

A total of 180 specimens were prepared and were randomly assigned to one of six experimental treatment groups (*n* = 30/group), all previously treated with 50 µm Al_2_O_3_ sandblasting: control (sandblasting only), 10‐MDP/silane primer (Clearfil Ceramic Primer Plus, Kuraray Noritake), universal adhesive containing 10‐MDP and HEMA (Single Bond Universal, 3M ESPE), phosphate methacrylate–based primer (Monobond N, Ivoclar Vivadent), methacrylate ethanol–based primer (YZAP Primer, Yller), and PMDM‐based primer (MZ Primer, Angelus).

For contact angle (wettability) and surface roughness (Sa) evaluations, 10 specimens per group were analyzed prior to and following primer treatment, totaling 60 specimens. The remaining 120 specimens were allocated for shear bond strength (SBS) testing, with 20 specimens per treatment group. Each group was further subdivided according to the type of resin cement used: dual‐cure self‐adhesive resin cement (RelyX U200, 3M) or dual‐cure conventional resin cement (Panavia V5, Kuraray) with 10 specimens for each resin cement.

The sandblasting with 50 µm Al_2_O_3_ was performed perpendicular to the sample surface, at a distance of 10 mm, for 15 s under 3 bar pressure. To standardize the sandblasting distance for all specimens, a specific apparatus (Basic Classic, Renfert) was used, along with a positioning device to hold the samples and nozzle support, keeping the samples fixed during the entire sandblasting process. Subsequently, specimens were washed with air–water spray for 30 s and dried with air jets.

After sandblasting with 50 µm Al_2_O_3_ particles, all zirconia specimens were ultrasonically cleaned in distilled water for 3 min and air‐dried. The bonding agents were applied according to the manufacturer's instructions, using a disposable microbrush to create a thin and uniform layer over the entire treated surface, followed by gentle air‐drying. Specifically, the 10‐MDP/silane primer was left for approximately 5 s before air‐drying for 5 s; the phosphate methacrylate–based primer was allowed to react for about 60 s before drying; the methacrylate ethanol–based primer remained for 3 min before air‐drying for 5 s; the PMDM‐based primer was left for 60 s before drying; and the universal adhesive containing 10‐MDP and HEMA was applied for 20 s and gently air‐dried for 5 s. All primers were applied as a single layer, ensuring complete coverage of the surface.

Wettability and surface roughness were evaluated both before and after primer application to assess the impact of surface treatments. Wettability (*n* = 10) was analyzed using the sessile drop technique with a goniometer (OCA‐20, DataPhysics Instruments). A 20 µL drop of Milli‐Q water was dispensed onto the zirconia surface, and contact angles were measured at two time points: initially and after 30 s. The change in contact angle (Δ*θ*) was calculated as Δ*θ* = *θ_f_ − θ_i_*, where *θ_f_* and *θ_i_* represent the final and initial mean values of the left and right contact angles, respectively. Measurements were performed in triplicate.

Surface roughness (*n* = 10) was assessed using a confocal laser microscope (LEXT OLS 4000, Olympus). Images of the surface were captured within a 2574 × 2577 µm field of view (scanning size at 107× magnification, 5× objective lens). The specimens were positioned on the microscope stage, and after identifying the area of interest, the surface was scanned. The software (LEXT 3D Measuring Laser Microscope OLS4000, Olympus) processed the images and automatically calculated the mean surface roughness (Sa) in micrometers. The data were exported as  .xls files, and surface topography images were also recorded for analysis.

To assess SBS, zirconia specimens were embedded in autopolymerizing acrylic resin (Resina Auto, TDV Dental) within PVC rings (1 cm height). Each sample was positioned at the center of the ring with the aid of a template to ensure alignment. Petroleum jelly was applied to the ring edges before pouring the acrylic resin. A parallelometer was used to maintain the correct positioning of the specimens during this process.

Resin composite cylinders (4 mm × 4 mm) were fabricated using a nano‐hybrid composite (Forma, Ultradent) inserted into a Teflon mold in two increments. The first increment was light cured for 20 s, and the second was covered with a polyester strip and a glass slide to ensure a flat surface and superficial polishing. After removing the glass slide, polymerization was completed through the polyester strip with two light exposures of 20 s each, using a curing unit (GrandValo, Ultradent) at 1000 mW/cm^2^.

Each composite cylinder was cemented onto a zirconia specimen after surface treatment according to the experimental groups. The resin cylinders were first cleaned in an ultrasonic bath with distilled water for 3 min. For specimens cemented with the dual‐cure conventional resin cement (Panavia V5, Kuraray), a primer containing 10‐MDP and silane (Clearfil Ceramic Primer Plus, Kuraray Noritake) was applied for 5 s and air‐dried. For the dual‐cure self‐adhesive resin cement (RelyX U200, 3M), the specimens were only air‐dried. The cementation procedure was performed using a parallelometer, ensuring the perpendicular positioning of each resin cylinder onto the zirconia surface under a constant 50 N load. The cement was mixed following each manufacturer's instructions, and the zirconia specimens were positioned at the base of the parallelometer. The resin cylinder was fixed to the vertical rod and placed perpendicularly on the zirconia surface. Excess cement was removed with a disposable microbrush (Stick, KG Sorensen), and light curing was performed using a LED curing unit (GrandValo, Ultradent) with an irradiance of 1000 mW/cm^2^. Polymerization was carried out for 20 s on each bonded surface, totaling 80 s per specimen. After this step, a glycerin‐based gel (Power Block, Maquira) was applied to prevent the oxygen inhibition layer and ensure complete polymerization of the resin cement.

Following cementation, the specimens were stored in distilled water at 37 ± 1°C for 24 h, followed by thermocycling for 10,000 cycles (simulating 1 year of clinical aging) using a thermal cycling machine (MSCT‐3, Marcelo Nucci). The samples were alternately immersed in water baths at 5°C and 55°C, with a dwell time of 30 s in each bath, following the protocol proposed by Gale and Darvell [20].

Upon aging, the SBS test was performed. The specimens were positioned in a shear test device (ISO/TR 11,405 standard) and tested using a universal testing machine (EMIC DL‐1000, Equipments and Systems) at a crosshead speed of 0.5 mm/min until failure. A chisel‐shaped loading blade applied a vertical shear force at the adhesive interface between the zirconia and the resin cylinder. A 50 kgf load cell recorded the debonding force, and SBS values (in MPa) were calculated using the formula SBS = *F*/*A*, where *F* is the force at failure and *A* is the bonding area. The values were tabulated for further statistical analysis.

After debonding, the zirconia surfaces were examined under a stereomicroscope (S8 APO, Leica Microsystems) at 30× magnification using a digital camera (DFC295, Leica Microsystems) to classify the failure mode according to the adhesive remnant index (ARI). The ARI scale ranges from 0 to 3:

0 – all cement remained on the zirconia surface;

1 – more than half of the cement remained on the zirconia surface;

2 – less than half of the cement remained on the zirconia surface;

3 – no cement remained on the zirconia surface.

The data were tested for normality and homogeneity of variance. As these assumptions were not met, a Wald test in a generalized linear model was performed for SBS, and a Wald test using generalized estimating equations (GEE) was applied for surface roughness (Sa) and wettability. All multiple comparisons were adjusted using the Bonferroni correction. A significance level of 5% was adopted. Statistical analyses were performed using IBM spss statistics 21.0.

## RESULTS

Surface roughness values before primer application were similar across groups, with mean values around 1.5 µm (95% confidence intervals [CIs] overlapping). After treatment, roughness increased in all conditions (Table 2). The universal adhesive containing 10‐MDP and HEMA showed the lowest post‐treatment value (3.6 µm; 95% CI 2.5–4.6), while the primers containing 10‐MDP with silane, phosphate methacrylate, or methacrylate in ethanol produced the highest roughness (ranging from 11.6 to 12.9 µm, with 95% CIs between 10.8 and 13.6). The sandblasted control presented an intermediate value (7.9 µm; 95% CI 7.1–8.8), as did the PMDM‐based primer (9.6 µm; 95% CI 8.1–11.2).

**TABLE 2:** Surface roughness (Sa, µm) and contact angle (°) values for zirconia specimens before and after primer application.

Experimental group	Roughness before primer mean ± SD (95% CI)	Roughness after primer mean ± SD (95% CI)	Contact angle before primer mean ± SD (95% CI)	Contact angle after primer mean ± SD (95% CI)
Control (Al_2_O_3_ sandblasting)	1.5 ± 0.0. (1.4–1.6)	7.9 ± 0.4 (7.0–8.7)*	79.8 ± 1.5 (76.8–82.8)	60.2 ± 2.1 (56.0–64.4)^a^
Primer (10‐MDP + silane)	1.5 ± 0.0 (1.4–1.5)	12.9 ± 0.4 (12.2–13.6)‡	80.6 ± 1.4 (77.9–83.3)	42.2 ± 1.8 (38.6–45.8)^b^
Universal adhesive (10‐MDP + HEMA)	1.6 ± 0.1 (1.5–1.7)	3.6 ± 0.5 (2.5–4.6)†	81.6 ± 1.6 (78.4–84.7)	42.1 ± 2.4 (37.4–46.9)^b^
Primer (silane + phosphate methacrylate)	1.5 ± 0.1 (1.4–1.6)	11.9 ± 0.5 (10.9–12.9)‡	79.9 ± 1.1 (77.8–82.1)	55.8 ± 2.0 (51.9–59.7)^a^
Primer (methacrylate in ethanol)	1.6 ± 0.1 (1.6–1.8)	11.6 ± 0.4 (10.9–12.4)‡	79.3 ± 1.5 (76.5–82.2)	50.2 ± 3.0 (44.3–56.2)^a^ ^b^
Primer (PMDM)	1.5 ± 0.0 (1.4–1.5)	9.6 ± 0.8 (8.1–11.2)†	82.2 ± 1.5 (79.4–85.1)	39.8 ± 1.7 (36.5–43.2)^b^

*Note*: Superscripts indicate significant differences among groups within each column (*p* < 0.05); symbols (*, †, ‡) for roughness; letters (^a^, ^b^, ^a^ ^b^) for contact angle.

Abbreviation: CI, confidence interval; SD, standard deviation.

Wettability analysis (Table 2) revealed that before primer application, contact angles were comparable across groups, ranging from 79° to 82°. After application, angles decreased with all primers. Sandblasted zirconia without primer showed the highest mean value (60.2°, 95% CI 56.0–64.4), followed by the silane‐based primer with phosphate methacrylate (55.8°, 95% CI 51.9–59.7). The methacrylate‐based primer in ethanol yielded an intermediate value (50.2°, 95% CI 44.3–56.2). The lowest contact angles were observed for the universal adhesive containing 10‐MDP and HEMA (42.1°, 95% CI 37.4–46.9), the primer containing 10‐MDP and silane (42.2°, 95% CI 38.6–45.8), and the PMDM‐based primer (39.8°, 95% CI 36.5–43.2).

SBS values for the two resin cements after application of the different primers are presented in Table 3. For the conventional dual‐cure resin cement, values ranged from 12.5 MPa (95% CI 9.1–13.9) for the sandblasted control to 18.3 MPa (95% CI 13.4–25.4) for the 10‐MDP/silane primer, while for the dual‐cure self‐adhesive resin cement, the lowest value was also observed for the sandblasted control (8.0 MPa; 95% CI 5.2–20.9) and the highest for the universal adhesive containing 10‐MDP and HEMA (16.5 MPa; 95% CI 12.4–19.9); no systematic differences were detected between the two resin cements. When results were analyzed irrespective of cement type (pooled across cements, Table 3), the highest bond strengths were obtained with the 10‐MDP/silane primer (17.9 MPa; 95% CI 15.9–19.9), the universal adhesive containing 10‐MDP and HEMA (17.0 MPa; 95% CI 14.9–19.0), and the phosphate methacrylate–based primer (17.1 MPa; 95% CI 15.1–19.1), whereas the PMDM‐based primer showed intermediate values (13.5 MPa; 95% CI 11.3–15.6) and the sandblasted control (10.1 MPa; 95% CI 7.8–12.4) together with the methacrylate ethanol–based primer (11.7 MPa; 95% CI 9.7–13.7) exhibited the lowest results.

**TABLE 3:** Shear bond strength (MPa) values observed for the bonding of dual‐core resin cements to sandblasted zirconia after different primer applications.

Experimental group	Conventional resin cement mean ± SD (95% CI)	Self‐adhesive resin cement mean ± SD (95% CI)	Pooled across resin cements mean ± SD (95% CI)
Control (Al_2_O_3_ sandblasting)	12.5 ± 2.1 (9.1–13.9)	8.0 ± 3.5 (5.2–20.8)	10.1 ± 2.7 (7.8–12.4)^a^
Primer (10‐MDP + silane)	18.3 ± 3.0 (13.4–25.4)	16.1 ± 2.9 (14.0–18.2)	17.9 ± 2.1 (15.9–19.9)^c^
Universal adhesive (10‐MDP + HEMA)	18.1 ± 2.4 (15.6–22.9)	16.5 ± 3.1 (12.4–19.9)	17.0 ± 2.0 (15.0–19.0)^b^ ^c^
Primer (silane + phosphate methacrylate)	16.3 ± 3.2 (11.4–22.9)	18.0 ± 3.4 (12.2–20.7)	17.1 ± 2.1 (15.1–19.1)^b^ ^c^
Primer (methacrylate in ethanol)	13.9 ± 3.1 (6.1–18.9)	16.0 ± 2.8 (10.1–18.3)	15.0 ± 2.95 (13.6–16.3)^a^
Primer (PMDM)	14.1 ± 3.5 (5.0–17.9)	10.1 ± 2.9 (7.2–12.4)	12.1 ± 3.21 (10.6–13.6)^a^ ^b^

*Note*: Comparisons among primers were performed on pooled data (*N* = 20 per primer). Different superscript letters indicate statistically significant differences among primers in the pooled analysis (*p* < 0.05). No significant differences were observed between resin cements (*p* = 0.858) or for the primer × resin cement interaction (*p* = 0.902).

Abbreviation: CI, confidence interval; SD, standard deviation.

**FIGURE 2:**
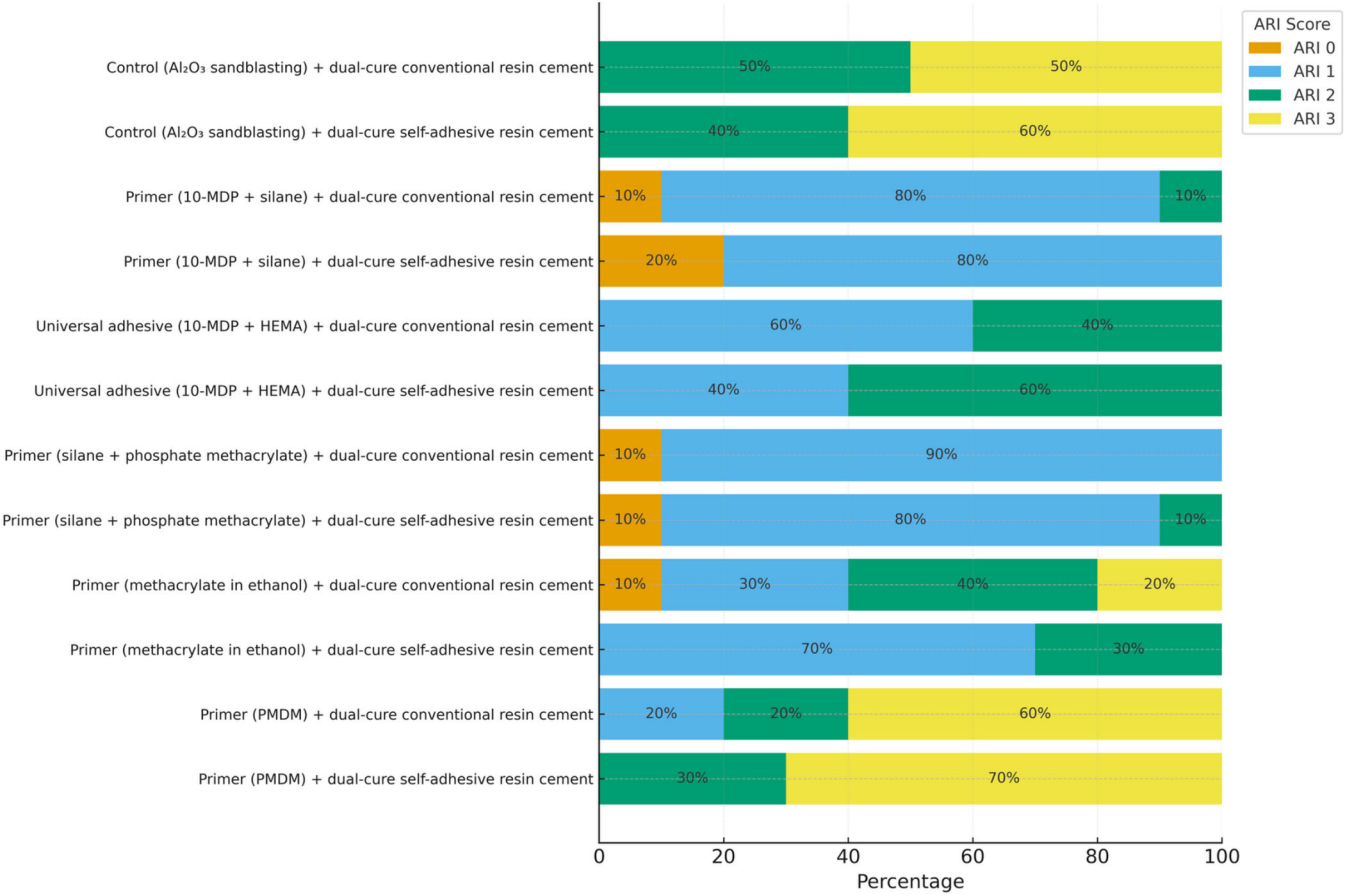
Percentage of adhesive remnant index (ARI) following artificial aging and shear bond strength testing of zirconia specimens.

The distribution of ARI scores is shown in Figure 2. The 10‐MDP/silane primer and the phosphate methacrylate–based primer predominantly resulted in ARI = 1 (80%–90%), whereas sandblasted zirconia without primer and the PMDM‐based primer exhibited higher proportions of ARI = 2 and ARI = 3, with up to 70% of specimens retaining large amounts of cement. The universal adhesive containing 10‐MDP and HEMA and the methacrylate ethanol–based primer showed intermediate distributions, mainly between ARI = 1 and ARI = 2. These tendencies were consistent regardless of whether dual‐cure conventional or self‐adhesive resin cement was used. Inter‐examiner agreement for ARI scoring was substantial (κ = 0.61).

## DISCUSSION

The interaction between zirconia ceramics and luting agents critically depends on the physicochemical characteristics of their surface, such as roughness and wettability [21]. In this study, different surface treatments applied to zirconia promoted significant changes in these properties, directly affecting the adhesive performance of restorations. The chemical composition of primers emerged as a key determinant of bond strength, independent of mechanical modifications. Analyses revealed that both the type of treatment and the treatment stage (pre‐ and post‐treatment) significantly influenced roughness values, contact angle, and bond strength after thermocycling. Therefore, both null hypotheses were rejected, as the surface treatments significantly influenced surface roughness, wettability, and bond strength, demonstrating that the chemical composition of primers and their combination with sandblasting directly affected zirconia adhesion. Among the tested protocols, sandblasting combined with 10‐MDP/silane or phosphate methacrylate–based silane primers yielded the highest bond strength and most favorable ARI values, highlighting the importance of functional monomers such as 10‐MDP for effective zirconia adhesion.

Modifications in zirconia surface topography play a decisive role in the quality of the adhesive interface, particularly with primers containing functional monomers such as 10‐MDP [18]. Most surface treatments significantly increased roughness, except for the universal adhesive containing 10‐MDP and HEMA, which showed minimal change. This lower roughness, due to the absence of mechanical conditioning, suggests that mere application without abrasive pretreatment may be insufficient for effective micromechanical retention. It is important to note that Single Bond Universal was originally developed for enamel and dentin, and its use on zirconia represents an off‐label application, which may partly explain its distinct performance. Nevertheless, its satisfactory performance can be partially attributed to the presence and concentration of 10‐MDP, which chemically interacts with zirconia surface oxides, and to its slightly acidic pH, which may enhance surface wetting and interaction. In contrast, sandblasting combined with chemical treatments, such as the 10‐MDP/silane and PMDM primers, induced pronounced increases in roughness, supporting the notion that the combination of mechanical and chemical strategies promotes more durable adhesion [22, 23]. Nevertheless, increased roughness alone does not always translate to higher bond strength, as chemical interactions can enhance adhesion even in the absence of pronounced mechanical changes [24].

Sandblasting with aluminum oxide particles creates micromechanical retentions that enhance primer anchorage, especially for MDP‐containing primers, increasing both contact area and surface energy of zirconia [13]. However, excessive abrasion may compromise fracture resistance, particularly in thin restorations [24]; thus, roughness data should be interpreted alongside wettability and bond strength for a clinically relevant assessment [18].

Wettability analysis showed significant reductions in contact angles across all groups. The PMDM primer, the universal adhesive containing 10‐MDP and HEMA, and the 10‐MDP/silane primer exhibited the lowest angles, reflecting higher surface affinity, whereas the control (sandblasting only) and phosphate methacrylate–based primer showed higher angles. These results indicate that primer composition, particularly functional monomers, is key to enhancing surface energy and adhesion. This effect is mainly due to 10‐MDP in the Clearfil Ceramic Primer and Single Bond Universal Adhesive, which forms stable chemical bonds with zirconia oxides, favoring wettability and monomer diffusion [25, 26, 27]. Although Single Bond Universal was not originally intended for ceramics, its 10‐MDP content explains its favorable performance. The Clearfil Ceramic Primer, combining silane and 10‐MDP, performed better than the control and Monobond N, since silane alone is less effective on zirconia [26, 28, 29]. Monobond N showed lower adhesion due to the absence of functional monomers with strong zirconia affinity, and without prior abrasive treatment, its bonding was insufficient [25, 27].

The SBS evaluation revealed that groups treated with primers combined with prior sandblasting, such as the 10‐MDP/silane primer and the phosphate methacrylate–based silane primer, exhibited the highest values. This performance is attributed to the synergy between mechanical activation via sandblasting and the chemical functionality of monomers, such as 10‐MDP in Clearfil Ceramic Primer Plus or phosphate and acidic methacrylate monomers in Monobond N, which form stable bonds with zirconia [12, 30, 31]. The universal adhesive containing 10‐MDP and HEMA also showed satisfactory results, supporting adhesion even without abrasive pretreatment. In contrast, the MZP primer, containing PMDM but lacking 10‐MDP, demonstrated lower bond strength comparable to the control [32]. Despite high surface roughness in the methacrylate‐based primer in ethanol, bond strength was intermediate and lower than with 10‐MDP/silane, phosphate methacrylate–based silane primer, or universal adhesive, indicating that surface topography alone does not ensure effective adhesion [33, 34].

The effect of resin cement formulation on bond strength was also analyzed after thermocycling, using dual‐cure conventional resin cement (Panavia V5) and dual‐cure self‐adhesive resin cement (RelyX U200). Although dual‐cure conventional resin cement showed a trend toward higher bond strength values, no statistically significant differences were found between the two cements. This suggests that while resin cement composition can influence bond durability to some extent, the choice of surface treatment—especially when involving MDP‐containing primers—is the predominant factor determining zirconia bonding efficacy [19, 35, 36].

The ARI analysis complemented the bond strength data. In groups with higher bond strength, the phosphate methacrylate–based silane primer and 10‐MDP/silane primer predominated with ARI = 1, indicating failure at the cement–zirconia interface, characteristic of strong bonds. The universal adhesive containing 10‐MDP and HEMA and the methacrylate‐based primer in ethanol showed mixed ARI = 1 and 2, consistent with intermediate performance. Meanwhile, the control and PMDM primer groups had more ARI = 2 and 3, associated with weaker bonds and cohesive or mixed failures. ARI serves as an indirect indicator of adhesive quality and failure mode, with lower values reflecting more effective clinical adhesion [37, 38].

It is worth noting that although MDP‐containing primers generally yield good results, there are relevant differences among commercial products. The present study demonstrates that the mere presence of the functional monomer does not guarantee superior performance; product formulation, application protocol, and compatibility with the luting system must also be considered [39]. Additionally, factors such as application technique and primer action time also influence adhesive performance [40, 41].

This study has some limitations. Bond strength was evaluated under controlled laboratory conditions, which may not fully reflect the clinical behavior of zirconia restorations over time. Thermocycling simulates intraoral temperature changes but does not capture complex mechanical and chemical oral effects. Clinically, the results reinforce the importance of proper zirconia surface treatment to ensure durable bonding with resin cements, with sandblasting combined with 10‐MDP–containing primers remaining the gold standard. Variability among commercial products, clinical conditions, and primer–cement compatibility highlights the need for customized protocols. Although manufacturers recommend primer use without abrasion, this was not tested and represents a limitation. Future studies should evaluate primers applied without mechanical treatment and incorporate more realistic aging simulations, including cyclic loading and exposure to biological fluids, to optimize surface treatments and validate long‐term zirconia–resin adhesion.

## AUTHOR CONTRIBUTIONS

**Conceptualization**: Otavio Marino dos Santos Neto. **Methodology**: Otavio Marino dos Santos Neto, Ingrid Carneiro Cavalcante Souto, Ana Paula Macedo, and Rossana Pereira de Almeida. **Resources**: Rossana Pereira de Almeida. **Data curation**: Ingrid Carneiro Cavalcante Souto and Ana Paula Macedo. **Formal analysis**: Ana Paula Macedo, Ingrid Carneiro Cavalcante Souto, and Otavio Marino dos Santos Neto. **Writing—original draft**: Otavio Marino dos Santos Neto and Ana Paula Macedo. **Writing–review and editing**: Rossana Pereira de Almeida, Otavio Marino dos Santos Neto, and Ana Paula Macedo. **Project administration**: Rossana Pereira de Almeida.

## CONFLICT OF INTEREST STATEMENT

None.
